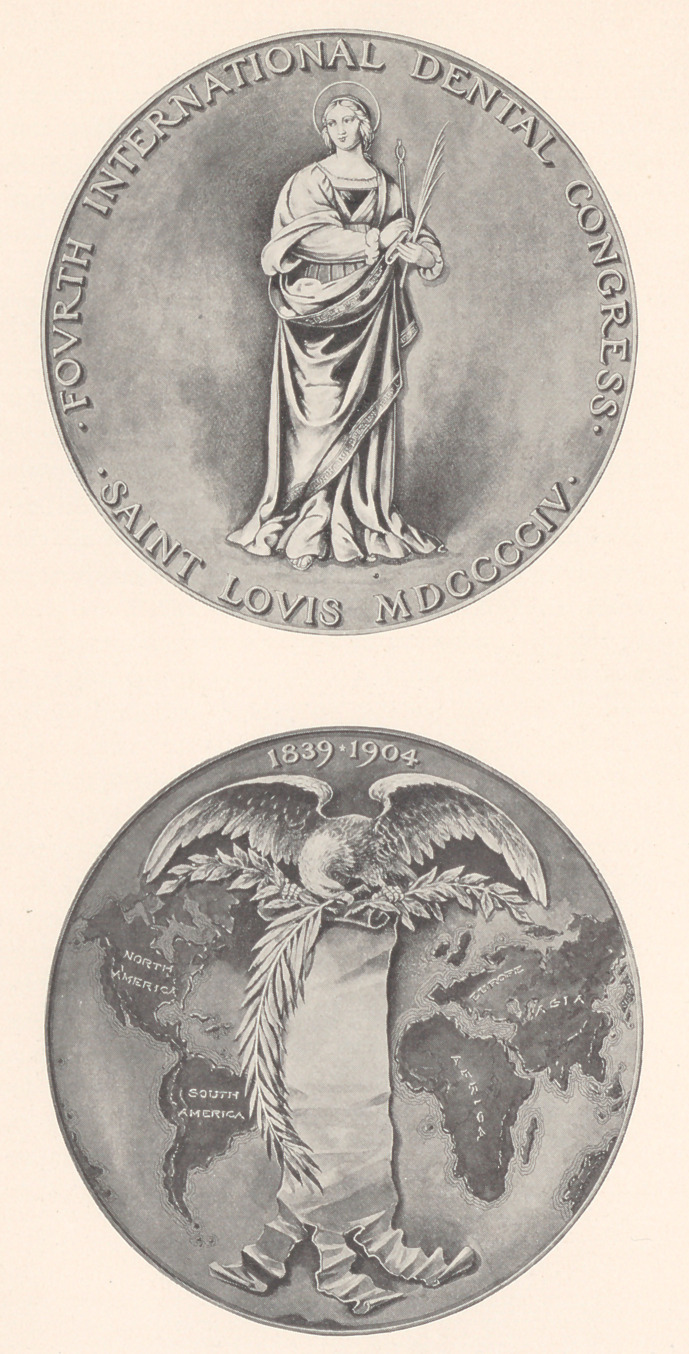# The Souvenir Medal of the Fourth International Dental Congress

**Published:** 1903-12

**Authors:** 


### THE SOUVENIR MEDAL OF THE FOURTH INTERNATIONAL DENTAL CONGRESS.

THE illustration subjoined represents the medal authorized by the Committee of Organization of the Fourth International Dental Congress, to be held next year in St. Louis, Mo., August 29 to September 3, inclusive.

The figure upon the obverse side is that of St. Apollonia, the so-called patron saint of dentistry. This seems not only appropriate, but is an artistic reproduction of the original picture. The committee having the matter in charge speak of the reverse side of the medal as "having had ample consideration, and it is such, we think, as should meet with general approval. The universality and international character of the Congress movement is typified by the continental divisions of the world. The associated dates at the top of the design are those which embrace the professional life-history of dentistry. Falling gracefully down between the continents is a scroll upon which is to be inscribed the names of the recognized fathers of dentistry in all countries, each national body being asked to nominate the name or names to represent the respective countries. The pose of the eagle represents the auspices under which the Congress is to be held, and the palm branch a tribute of honor on behalf of the American profession to the fathers of dentistry.

"The execution of the dies will be intrusted to the most expert die-sinker in America. The design will be in high relief, and the medal will be struck in bronze, and will be about two and one-half inches in diameter. It will be a finished work of art in all respects, and an attractive and interesting souvenir of the great meeting which it typifies.

"The medal will be supplied only to those who make application for it in advance of the Congress, as the number struck will be limited to the number subscribed for. The price of the medal without a case has been fixed at five dollars. Cases for the medal will be furnished at prices corresponding with their character and quality."

The committee having this matter in charge are to be congratulated in having been able to produce such an artistic and at the same time a valuable memento of the Congress. It is imagined there will be some difficulty in selecting the father of dentistry in each country to be inscribed on the scroll on the reverse side. The decision must rest, in this country, between Harris and Hayden. The choice will be difficult, for both were almost equally active in the development of the intellectual side of the profession.

## Figures and Tables

**Figure f1:**